# Prevalence of p24 antigen among a cohort of HIV antibody negative blood donors in Sokoto, North Western Nigeria - the question of safety of blood transfusion in Nigeria

**DOI:** 10.11604/pamj.2014.18.174.3449

**Published:** 2014-06-22

**Authors:** Erhabor Osaro, Ndakotsu Mohammed, Isaac Zama, Abdulrahaman Yakubu, Ikhuenbor Dorcas, Aghedo Festus, Ibrahim Kwaifa, Ibrahim Sani

**Affiliations:** 1 Department of Haematology and Transfusion Medicine, Faculty of Medical Laboratory Science Usmanu Danfodiyo University, Sokoto, Nigeria; 2 Department of Haematology and Blood Transfusion Usmanu Danfodio University Teaching Hospital Sokoto

**Keywords:** P24 antigen, blood donors, blood transfusion, safety, Nigeria

## Abstract

**Introduction:**

Blood transfusions remain a substantial source of HIV in SSA particularly among children and pregnant women. Aims and objectives: This aim of this retrospective study was to investigate the prevalence of p24 antigen among HIV antibody seronegative blood donors in Sokoto, North West Nigeria.

**Methods:**

A total of 15,061 HIV antibody negative blood donors with mean age and age range (29.2 ± 8.18 and 18-50 years) were screened for p24 antigen between January 2010 to July 2013 using the Diapro Diagnostic immunoassay kit for P24 antigen (King Hawk Pharmaceuticals Beijing China).

**Results:**

The overall prevalence of p24 antigen among the HIV antibody negative donors sample was 5.84%. The yearly prevalence was 9.79, 8.12, 2.7 and 2.84% respectively in 2010, 2011, 2012 and 2013. Of the total number of blood donor tested, 14,968 (99.38%) were males while 93 (0.62%) were females. The prevalence of P24 antigen was significantly higher among male blood donors 873 (5.8%) compared to females 7(0.05%), (p= 0.001). P24 positivity was significantly higher among blood group O blood donors compared to A, B and AB donors (494 (3.29%) compared to 184 (1.89%), 196 (1.30%) and 6 (0.04%)) respectively, p = 0.001). The prevalence of P24 antigen was significantly higher among Rhesus positive blood donors compared to Rhesus negative (807 (5.36%) versus 73 (0.48%), p =0.001).

**Conclusion:**

Blood transfusion in Nigeria is associated with increased risk of HIV transmission. There is the urgent need to optimize the screening of blood donors in Nigeria by the inclusion of p24 antigen testing into the blood donor screening menu. The Nigerian government urgently need to adopt the WHO blood safety strategies to reduce the risk of transmission of HIV through blood transfusion.

## Introduction

The World Health Organization has estimated that there were 40 million people were infected with human immunodeficiency virus (HIV) globally at the end of 2001, and the majority of them were in developing countries [1]. The countries affected the most are economically poor and therefore unable to afford expensive diagnostic and monitoring tests. Blood transfusion is an essential part of modern medical care. Inadequate and unsafe blood supply causes avoidable deaths and transmits infectious diseases, including HIV. Transfusion of blood infected with HIV is one of the most effective modes of transmission of the virus. The risk of acquiring HIV infection following transfusion with HIV-positive blood has been estimated to be as high particularly in sub Saharan Africa (SSA). The use of HIV antibody -based test for the screening of blood donor is sub optimal. Evidenced- based data and best practices from the developed world indicates that implementing evidenced based national testing and donor selection algorithm that establishes the use of tests capable of detecting donation in the window phase of HIV infection (P24 and NAT) is the only way forward to reducing the potential of transmitting HIV infection through blood transfusion [2]. The HIV antibody test offers the advantages of simplicity and cost effectiveness for verifying infection, but it is less than perfect because of the possibility of transfusing antibody negative unit from a donor in the window phase of HIV infection. Newer technologies exist that can contribute to an accurate diagnosis, assist in monitoring the response to therapy, and can be used to effectively predict disease outcome. Viral isolation through viral culture, nucleic acid tests to detect viral RNA, and tests to detect p24 antigen can be used to demonstrate virus or viral components in blood, thereby verifying infection and potentially reducing the risk of transfusion of blood in the window phase of HIV infection. The p24 antigen assay measures the viral capsid (core) p24 protein in blood that is detectable earlier than HIV antibody during acute infection. It occurs early after infection due to the initial burst of virus replication and is associated with high levels of viremia during which the individual is highly infectious but may be antibody negative [2]. There is high rates of HIV in SSA countries and this continue to present a substantial challenge for blood services in recruiting and retaining safe blood donors. In sub-Saharan Africa, transfusion-transmitted human immunodeficiency virus (HIV) infection persists, particularly among women with pregnancy and haemorrhage –related anaemia and children with malaria –related anaemia who are the major recipient of blood transfusions [3]. In 2008, out of approximately 92 million blood units donated worldwide, only an insignificant 4 million (4.3%) were donated in sub-Saharan Africa, a continent that account for approximately 12% of the global population and where blood collections historically have been inadequate to meet clinical demand and inappropriate clinical use of blood [4]. Collections of donor blood is primarily from hospital-based services that relied on family members and commercial remunerated donors. Family replacement donors are usually under undue pressures to donate, might not reveal behavioural risks for HIV during donor selection and are typically at a greater risk for HIV infection than voluntary non-remunerated donors [5]. Blood transfusion services in Nigeria screen donated blood for markers of HIV infection using antibody based rapid test. The HIV antibody test offers the advantages of simplicity and cost effectiveness for verifying infection, but it is less than perfect because of the possibility of transfusing antibody negative donor unit in the window phase of HIV infection. There is paucity of data on the prevalence of p24 antigen among HIV antibody negative donors in Nigeria. HIV testing in the country is sub- optimally based on the detection of antibody to HIV virus. The risk of transfusing donor unit in the window phase of infection is unknown. Therefore, the aim of this case study was to investigate the prevalence of p24 antigen among HIV antibody negative blood donors in Sokoto, North Western, Nigeria. Evidence-based data generated will help in the formulation of policy to improve the safety of blood and blood product as well as optimize the quality of blood transfusion service delivery in Nigeria.

## Methods

### Study participants

This present retrospective study included a total of 15,061 blood donors who were screened at the blood transfusion unit of the Usmanu Danfodiyo University Teaching Hospital for HIV using antibody based rapid HIV test kits (Abbott Determine and STAT-PAK) between January 2010 to July 2013. All certified unreactive samples from conventional HIV rapid antibody-based test kits were tested for presence of P24 antigen. Ethical clearance was obtained from the ethical committee of the Usmanu Danfodiyo University Teaching Hospital in Sokoto, North Western Nigeria. Verbal informed consent is routinely obtained from all blood donors visiting the blood transfusion unit and Donors are offered pre and post test counselling.

A total of 15,061 blood donors were screened for HIV from January 2010 to July 2013. All certified unreactive samples from conventional antibody-based HIV rapid test kits (Abbott Determine and HIV 1/2 Stat-Pak) were tested for p24 antigens. The rapid antibody based kits are part of a multi-test algorithms designed for the statistical validation of rapid HIV test results. Abbott determine HIV test kit (Abbott, Abbott Park, Ill.), detects HIV type 1 (HIV-1) and HIV-2 antibodies within 15 minutes by using 50 µl of serum or plasma. The Chembio HIV 1/2 STAT-PAK^™^ Assay (Chembio Diagnostic, Medford, USA) is a single-use immunochromatographic test for the detection of antibodies to Human Immunodeficiency Virus Type 1 (HIV-1) and Type 2 (HIV-2) in finger stick whole blood, venous whole blood, serum or plasma specimens. The Chembio HIV 1/2 STAT-PAK^™^ assay is intended for use as a point- of-care test to aid in the diagnosis of infection with HIV-1 and HIV-2. This test is suitable for use in multi-test algorithms designed for the statistical validation of rapid HIV test results. All the 2 rapid diagnostic test meet the requirement for an ideal test for the rapid diagnosis of HIV infection; rapid, inexpensive, highly sensitive and specific, easy to perform; results easy to interpret; test should be able to be stored at room temperature with a long shelf life; and no additional equipment or ancillary supplies should be required to perform the test [7]. P24 antigen testing was carried out using the Diapro Diagnostic immunoassay kit for P24 antigen (King Hawk Pharmaceuticals Beijing China). All initially positive samples were confirmed using the Bioprobes Sri P24 immunoassay kit (Bioprobes, Milano, Italy).

### Study area

This present research work was carried out at the Haematology and Blood Transfusion unit of Usmanu Danfodiyo University Teaching Hospital in Sokoto in the North West geo-political zone of Nigeria. The hospital is a 500-bed teaching hospital and a centre of excellence in the rendering of specialist medical care to people in Sokoto metropolis and neighbouring states of Zamfara and Kebbi State. Sokoto State is located in the extreme North Western part of Nigeria near to the confluence of the Sokoto River and the Rima River. With an annual average temperature of 28.3 °C (82.9 °F), Sokoto is, on the whole, a very hot area. However, maximum daytime temperatures are for most of the year generally under 40 °C (104.0 °F). The warmest months are February to April when daytime temperatures can exceed 45 °C (113.0 °F). The rainy season is from June to October during which showers are a daily occurrence. There are two major seasons, wet and dry which are distinct and are characterized by high and low malarial transmission respectively. Report from the 2007 National Population Commission indicated that the state had a population of 3.6 million [6].

### Statistics

Statistical analyses were conducted using SPSS (version 11) software. Comparisons between populations were made using the Student′s t-test for parametric data and the Mann-Whitney test for non-parametric data. An alpha value of <0.05 denoted a statistically significant difference. Correlation was compared using a version of linear regression analysis.

## Results

A total of 15,061 blood donors were screened for HIV from January 2010 to July 2013 (Table 1). The mean age and age range of blood donors was 29.2 ± 8.18 and 18-50 years respectively. All certified unreactive samples from conventional antibody-based HIV rapid test kits (Abbott Determine and Stat Pak) were tested for presence of P24 antigen. P24 antigen testing was carried out using the Diapro Diagnostic immunoassay kit for P24 antigen (King Hawk Pharmaceuticals Beijing China). All initially positive samples were confirmed using the Bioprobes Sri P24 immunoassay kit (Bioprobes, Milano, Italy). Out of the 15,061 seronegative donors, 880 (5.84%) were positive while 14,181 were negative (94.16%) with P24 assay. The prevalence of P24 positive result on antibody negative donors sample was 9.79, 8.12, 2.7 and 2.84% in 2010, 2011, 2012 and 2013 respectively. Of the total number of blood donor tested, 14,968 (99.38%) were males while 93 (0.62%) were females. The prevalence of P24 antigen was significantly higher among antibody negative male donors 873 (5.8%) compared to females 7(0.05%), (p= 0.001) (Table 2). P24 positivity was compared based on the ABO and Rhesus blood groups of blood donors. P24 positivity was significantly higher among blood group O blood donors compared to A, B and AB donors (494 (3.29%) compared to 184 (1.89%), 196 (1.30%) and 6 (0.04%) respectively, p = 0.001) (Table 3). The prevalence of P24 antigen was significantly higher among Rhesus positive donors compared to Rhesus negative (807 (5.36%) versus 73 (0.48%), p =0.001) (Table 4).

**Table 1:** Shows the yearly positive P24 Antigen screened blood

Year	Number of Donors Tested	Number (%) P24 positive	Number (%) P24 negative	p-value
**2010**	2941	288 (9.79%)	2653 (90.21%)	0.001
**2011**	4,815	391 (8.12%)	4,424 (91.88%)	
**2012**	4,703	127 (2.70%)	4576 (97.30%)	
**2013**	2,602	74 (2.84%)	2,528 (97.16%)	

**Table 2:** Shows the positive P24 Antigen screened blood among gender.

Gender	Total Number (%) screened	Number (%) p24 antigen positive	Number (%) p24 antigen negative
**Male**	14,968 (99.38%)	873 (5.80%)	14,095 (93.59%)
**Female**	93 (0.62%)	7 (0.05%)	86 (0.57%)
**Total**	15,061 (100%)	880 (100)	14181 (100%)

**Table 3:** The prevalence of p24 positivity based on the ABO blood groups of blood donors

ABO blood group	Number (%) tested	Number (%) 24 Antigen Positive	Number (%) 24 Antigen Positive
**A**	3023 (20.07%)	184 (1.89%)	2839 (18.85%)
**B**	3375 (22.41%)	196 (1.30%)	3179 (21.12%)
**AB**	125 (0.83%)	6 (0.04%)	119 (0.79%)
**O**	8538 (56.69%)	494 (3.29%)	8044 (53.41%)
**Total**	15061 (100%)	880 (5.84%)	14181 (94.16%)

**Table 4:** The prevalence of p24 positivity based on the Rhesus blood groups of blood donors

Rhesus (Rh) blood group	Number (%) tested	Number (%) 24 Antigen positive	Number (%) 24 Antigen negative
**Rh D positive**	14,056 (93.33%)	807 (5.36%)	13,249 (87.97%)
**Rh D negative**	1005 (6.67%)	73 (0.48%)	932 (6.19%)
**Total**	15061 (100%)	880 (5.84%)	14181 (94.16%)

## Discussion

Compared to most developed countries of the world, the risk of human immunodeficiency virus (HIV) transmission by transfusion of blood and blood products is extraordinarily high in SSA. High level of blood safety in developed countries has been accomplished by successive refinement in donor screening and testing procedures for the detection and inactivation of different infectious agents in blood and blood products. The introduction of P24 antigen testing and NATs in European blood centres has improved output to detect donations from individuals in the very early stages (window phase) of infection [8, 9]. In most settings in Nigeria, donor screening for HIV is still entirely antibody based. In this present study we observed that 5.84% of donors certified HIV negative by antibody based test were positive for p24 antigen. The p24 antigen test has become an important test in determining the presence of viral antigen in individuals declared seronegative by an antibody- based test [10]. Our finding is in agreement with previous report in the United States of America which indicated that testing for HIV p24 antigen can potentially shorten the seronegative window to less than 20 days [11]. The detection rate of donors who are positive for p24 antigen but negative by antibody- based test was observed to be several cases per year among 12 million annual blood donations. Similarly observation from Argentina, a country where blood donor HIV antibody detection has been mandatory and p24 antigen screening is recommended, indicates that out of a total of 30,132 consecutive donations screened for HIV, a total, 0.3623% of samples were repeatedly reactive. Only one donor who tested non-reactive for HIV Ab, was repeatedly reactive for p24 Ag [12]. Our finding is at variance with previous report in Saudi Arabia which tested 24,654 blood donors but failed to detect a single p24 positive case [13]. The reasons for this observation may be due to the fact that the prevalence of HIV among people in Saudi Arabia is significantly low compared to Nigeria where the prevalence is high. Secondly blood donation in Saudi Arabia is based predominantly on voluntary non-remunerated blood donors unlike in Nigeria where a significant proportion of blood donors are high risk commercial remunerated and family replacement. Thirdly Saudi Arabia has an optimally run national blood transfusion service unlike in Nigeria where the national blood transfusion programme is still in the elementary phase of implementation.

Our finding from this study is an indication that blood transfusion in Nigeria is unsafe because there is a high risk of HIV transmission through the transfusion of blood supposedly screened seronegative by prevalent antibody- based test. This observation is at variance with observation from other countries where additional laboratory testing has been found to potentially shorten the seronegative window and produce a reduction in the risk of HIV transfusion through blood transfusion. HIV p24 antigen testing was required for all US blood donations [14]. In Canada, the US and in most developed countries, all donations are tested for the presence of antibodies to HIV-1 and -2, HCV, HTLV, syphilis, hepatitis B surface antigen (HbsAg), p24 antigen (HIV) and also for HIV and HCV nucleic acids. The introduction of new and improved screening tests for transfusion-transmissible diseases has led to remarkable improvement in the safety of the blood supply, with substantial shortening of the window period for HIV, HCV, and HBV infections [15]. Kenya began implementing measures to reduce transfusion-transmitted infections (TTIs) in its national blood supply in 2001. Donations are voluntary and non-remunerated, and donors undergo a health exam and answer a behavioral risk screening questionnaire. Donations are also screened using fourth-generation p24 antigen and HIV 1 and 2 antibody tests (ELISA) [16, 17]. In 2005, the South African National Blood Service introduced individual-donation (ID) nucleic acid test (NAT) screening for human immunodeficiency virus (HIV) RNA. One-year ID-NAT screening of 732,250 donations interdicted 16 HIV, 20 HBV, and 1 HCV window phase donations [18].

In most developed countries of the world, the risk of human immunodeficiency virus (HIV) transmission by transfusion of blood and blood products is extraordinarily small. This level of blood safety has been accomplished by successive refinement in donor screening and testing procedures for the detection and inactivation of different infectious agents in blood and blood products. In USA and European blood centres, the introduction of nucleic acid techniques (NAT) in blood banks for the detection of HIV and hepatitis C virus (HCV) has meant a great advance in decreasing the residual risk of HIV/HCV transmission by blood transfusion [19, 20]. Communities in Africa faces several enduring challenges; chronic blood shortages, high prevalence of transfusion-transmissible infection, absence of national blood transfusion service, recruitment and retention of voluntary non –remunerated donors, family replacement and commercial blood donation, inadequate use of pharmacologic and non-pharmacologic alternatives to allogenic blood. Approximately 80 percent of the world′s supply of safe blood goes to 20 percent of the population, mostly in developed countries. There is a chronic shortage of safe blood in sub-Saharan Africa where haemorrhage (from road traffic accident, communal clashes, post-surgical, post and antepartum) accounts for up to 44 percent of maternal deaths and approximately half of transfused blood goes to pregnant women with anaemia and pregnancy related complications as well as children under five years of age with anaemia due to malaria or malnutrition. Africa has the lowest blood donation rate per capita in the world and blood donation are almost entire family replacement and commercial remunerated donors based. These groups of donors are at greater risk for transfusion-transmissible diseases than are voluntary non-remunerated donors (VNRD). In addition, blood shortages may lead to release of blood for transfusion before testing is completed, further adding to the potential for disease transmission. These problems are exacerbated in regions with high endemic rates of transmissible diseases. It is important for countries in Africa to devise strategies aimed at recruiting potential donors who are least likely to harbour transfusion-transmissible infectious agents. There are several reasons for the high risk of transfusion of blood in the window phase of infection in SSA. Despite recommendations that all blood donors should be voluntary and non-remunerated, replacement donors are common throughout SSA. National blood transfusion programs are not available and even when available are sub optimally managed. The primary steps of setting up a national blood transfusion programme includes; the enactment of a national policy for the blood transfusion service with time-bound programmes, a centrally coordinated, structured and organized blood transfusion service for a country/state under a defined authority, a blood transfusion service based on an organized voluntary blood donor programme, screening blood for transfusion-transmissible infections (TTI'S) appropriate to the region, appropriate and evidence based use of available blood and blood products and employment and retention of qualified personnel to head and manage the blood transfusion service [21]. In many countries in SSA, majority and sometimes none of these steps are in place. There is lack of political will and open-mindedness to innovative ways to improve supply and safety of blood from voluntary donors. The resultant effect of this failure in the stewardship of blood and blood products is that blood transfusions remain a substantial source of HIV in SSA especially among women with pregnancy-related complications and children with malaria and malnutrition –associated anaemia [22]. The incidence of transmission transmissible infections is generally high and transfusion of blood in the window phase of HIV infection continues to be prevalent because of reliance on antibody -based test for donor screening and non- inclusion of more sensitive p24 antigen test and nucleic acid testing in their donor testing menu. Another reason why majority of donors in Africa may be in the window phase of infection is that a significant proportion of these donors are commercial remunerated donors. Previous reports in most countries in SSA have indicated a high prevalence of transfusion- transmissible infections among commercially remunerated blood donors [23, 24]. Commercial remunerated donors often come from the poorest sectors of the economy, may be poor in health, are more likely to give blood more often than recommended, are at a higher risk of being undernourished and having a transfusion-transmissible infection from high risk behaviours like maintenance of multiple sex partners, intravenous drug abuse and unprotected sexual intercourse [25–27].

There seems a disparity between developed countries that rely on high-performance technology to guarantee safe supplies of donated blood and resource-poor countries particularly in SSA facing many obstacles to safe blood provision. Namely those obstacles include limited budget; the high prevalence of HIV, prevalence of commercial and family replacement donors, challenge of recruitment and retention of voluntary remunerated donors, reliance on the use of cheap rapid antibody- based tests for screening of transfusion transmissible infections rather than striving for more sensitive and high technology-based testing [28–30]. Following infection with HIV, the sequence of appearance of biologic markers in serum to facilitate the identification of infection often follow the chronologic order; viral RNA, p24 antigen, and anti-HIV antibody. About 2 weeks after infection, viremia is thought to increase exponentially and then decline to a steady-state level as the humoral and cell-mediated immune responses control HIV replication. Antibody remains detectable throughout infection except during the window phase of infection, whereas p24 antigen characteristically appears early and late during infection. A combination of an antibody based test and P24 antigen based test should be the minimum requirement for testing of blood donor for HIV. However, the main objective of HIV testing in donor is to eliminate the risk of HIV transmission through blood transfusion. The clinically significant time interval between infection and detection of antibodies (window period) is vital. Effort should made to reduce the risk of transfusing donor blood within this period. This period is characterized by a sero negative result from an antibody based test, detectable antigenemia (p24) and viremia (as measured by RNA). It does seem that the way forward in achieving a zero tolerance in HIV transmission through blood transfusion is to screen donors with a combination of an antibody based test, P24 antigen test and nucleic acid (NAT) testing. Tests for viral nucleic acid have been introduced to further reduce the risk of transfusion of donor blood during the window phase of HIV infection in most developed countries, but require sophisticated technology and dedicated, well-trained personnel [31–33]. NAT costs 3 to 10 times more than ELISA. There is need for low-resource countries to carefully weigh the advantages and costs of using NAT. However a previous study to estimate the number of window period infections entering the Kenyan blood supply investigated over 12,000 antibody and p24 negative specimens from six national collection centres for HIV using NAT. NAT retesting found no additional HIV infections, indicating that HIV antibody and p24 antigen screening can significantly reduce HIV in the nation′s blood supply, even in a setting with a generalized HIV epidemic [16].

We compared the prevalence of p24 positivity based on the gender of the blood donors. P24 positivity was significantly higher among male blood donors compared to females. Our finding is at variance with previous reports in Nigeria which found the prevalence of HIV higher among females compared to males [34, 35]. The small population of females among the donor population studied may also have influenced this finding.

We compared the prevalence of p24 positivity based on the ABO and the Rhesus D blood groups of blood donors. P24 positivity was higher among group O and Rhesus positive blood donors compared to non-O donors and Rhesus negative blood donors. Certain diseases have been associated with certain ABO blood groups. Our finding is consistent with a previous reports in India which observed that HIV infection was more common among adults and paediatrics who are blood group O Rh positive [36, 37]. Similarly studies carried out in Enugu by Nneli and colleagues [38] and in Adamawa State by Abdulazeez and colleagues [39] indicated that HIV positivity was more predominant among group O subjects. Similarly, a study to investigate the distribution of ABO and Rhesus blood group types in 984 randomly selected human T lymphotropic virus-1(HTLV-1)-infected blood donors observed that donors who are AB+ have increased risk of HTLV-1 infection [40]. The reason for this predisposition may be genetic or it may also be due to the fact that blood group O is the most prevalent ABO blood groups among blacks and Caucasians.

## Conclusion

The Nigerian government must urgently adopt WHO blood safety strategies for resource-limited settings. There is the need to optimize the donor selection and testing algorithm. HIV screening of donors must become based on a combination of antibody-based test and p24 antigen test coupled with a stringent blood donor selection algorithm. Previous report indicates that except for adding HIV-p24 screening, adding other tests such as nucleic acid amplification testing (NAT) to HIV-antibody screening displayed incremental cost-effectiveness ratios greater than the WHO/World Bank specified threshold for cost-effectiveness particularly for developing countries [40].
